# Clinical endoscopic management and outcome of post-endoscopic sphincterotomy bleeding

**DOI:** 10.1371/journal.pone.0177449

**Published:** 2017-05-17

**Authors:** Wei-Chen Lin, Hsaing-Hung Lin, Chien-Yuan Hung, Shou-Chuan Shih, Cheng-Hsin Chu

**Affiliations:** 1 Division of Gastroenterology, Department of Internal Medicine, Mackay Memorial Hospital, Taipei, Taiwan; 2 Mackay Medicine, Nursing and Management College, Taipei, Taiwan; University Hospital Llandough, UNITED KINGDOM

## Abstract

Post-endoscopic sphincterotomy bleeding is a common complication of biliary sphincterotomy, and the incidence varies from 1% to 48%. It can be challenging to localize the bleeder or to administer various interventions through a side-viewing endoscope. This study aimed to evaluate the risk factors of post-endoscopic sphincterotomy bleeding and the outcome of endoscopic intervention therapies. We retrospectively reviewed the records of 513 patients who underwent biliary sphincterotomy in Mackay Memorial Hospital between 2011 and 2016. The blood biochemistry, comorbidities, indication for sphincterotomy, severity of bleeding, endoscopic features of bleeder, and type of endoscopic therapy were analyzed. Post-endoscopic sphincterotomy bleeding occurred in 65 (12.6%) patients. Forty-five patients had immediate bleeding and 20 patients had delayed bleeding. The multivariate analysis of risk factors associated with post-endoscopic sphincterotomy bleeding were liver cirrhosis (P = 0.029), end-stage renal disease (P = 0.038), previous antiplatelet drug use (P<0.001), and duodenal ulcer (P = 0.023). The complications of pancreatitis and cholangitis were higher in the bleeding group, with statistical significance. Delayed bleeding occurred within 1 to 7 days (mean, 2.5 days), and 60% (12/20) of the patients received endoscopic evaluation. In the delayed bleeding group, the successful hemostasis rate was 71.4% (5/7), and 65% (13/20) of the patients had ceased bleeding without endoscopic hemostasis therapy. Comparison of different therapeutic modalities showed that cholangitis was higher in patients who received epinephrine spray (P = 0.042) and pancreatitis was higher in patients who received epinephrine injection and electrocoagulation (P = 0.041 and P = 0.039 respectively). Clinically, post-endoscopic sphincterotomy bleeding and further endoscopic hemostasis therapy increase the complication rate of pancreatitis and cholangitis. Realizing the effectiveness of each therapeutic modalities and appropriate management of different levels bleeding are important.

## Introduction

Endoscopic sphincterotomy (ES) is the cornerstone of therapeutic endoscopic retrograde cholangiopancreatography (ERCP) for accessing the biliary and pancreatic ductal systems. Bleeding is one of the most frequent complications following ES [1]. The incidence varies from 1% to 48% depending on what definition is applied, specifically whether bleeding during the procedure is included [1–4]. It varies between self-limiting and life-threatening bleeding and is occasionally associated with a considerable mortality rate of 0.3% [3]. The timing of delayed post-ES bleeding may be immediate or up to 10 days following ES [2, 5]. A small amount of post-ES bleeding is common and most often resolves spontaneously [1]. Thus, endoscopic therapy was suggested to be undertaken for the treatment of “endoscopically significant” immediate bleeding or “clinically significant” delayed bleeding [1]. However, some cases present with significant bleeding that requires blood transfusions and urgent endoscopic intervention, such as injection, balloon tamponade, thermal, and mechanical methods—alone or in combination [6–9]. If refractory bleeding occurs, repeated endoscopic hemostatic therapy, angiographic embolization, or surgery is required [3, 4].

Owing to the tangential approach in these patients, the bleeding could not be handled easily with side-viewing endoscope and this urgent procedure could not be practically performed by the gastroenterologists experienced in ERCP each time. Furthermore, bleeding that obscures the visual field makes identification of the precise location in delayed bleeding impossible. The complications of pancreatitis, cholangitis, and cholecystitis associated with endoscopic hemostasis were higher in patients who underwent endoscopic hemostasis than in patients who did not undergo endoscopic hemostasis [10].

There are a few series that specifically describe effectiveness and complication of endoscopic hemostasis for post-ES bleeding [1, 7, 10, 11]. Therefore, the primary endpoint of this study was to evaluate the risk factors of post-ES bleeding. The secondary endpoint was to evaluate the efficacy and outcome of different endoscopic intervention therapies for post-ES bleeding.

## Materials and methods

### Patients selection and data collection

Five hundred thirteen patients who underwent ES at Mackay Memorial Hospital, Taipei Medical Center, between January 2011 and August 2016, were enrolled. All ES procedures were performed by four experienced endoscopists (experienced endoscopist as a specialist in ERCP with a minimum of 3 years post-training experience), and the decision to perform sphincterotomy was based on clinical, endoscopic, and radiologic findings. None of the participants used anticoagulants or antiplatelet agents from 3 days before or until 3 days after ERCP. Prophylactic antibiotic or rectal indomethacin was not prescribed in any of the patient. This study was approved by the Institutional Review Board of Mackay Memorial Hospital (reference number: 17MMHIS024) and waived the requirement for informed consent because the current study was retrospective in design. Patient information was anonymized and de-identified prior to analysis.

Sphincterotomy was performed with a side-viewing duodenoscope (JF-260V, Olympus, Tokyo, Japan) and pull-type sphincterotome (TRUEtome; Boston Scientific, Spencer, IN, USA). ES was performed using standard technique. After deep cannulation, the papillotome was retracted until approximately one-third of the wire remained within the papilla. In six difficult cases of deep cannulation, precut sphincterotomy was carried out using a pull-type sphincterotome. A feedback-controlled generator was used (Erbe, ICC350; Erbe, Tubingen, Germany), and blended current (cut/coagulation = 1:1) at a setting of 30 W was applied. ES was made toward the 11- and 1-o’clock direction, in a stepwise manner.

Clinical and laboratory parameters before and after ERCP were analyzed to identify the risk factors and outcome of post-ES bleeding. Patient factors included age, sex, biochemistry, comorbidities, endoscopic diagnosis, occurrence of immediate or delayed post-ES bleeding, severity of bleeding, endoscopic features on delayed bleeding, and type of endoscopic therapy.

### Endoscopic therapy

If post-ES bleeding occurred, endoscopic therapy for post-ES bleeding was performed using a side-viewing or end-viewing endoscope, with the type depending on the endoscopist’s discretion. Epinephrine was either injected (1:10,000 dilution; 3–20 mL) or irrigated (1:50,000 dilution; 30–50 mL) around the bleeder at the sphincterotomy site until bleeding was controlled. Thermotherapy was attempted with the sphincterotome wire or heater probe, and the power setting was on forced coagulation mode of 30 W. Balloon tamponade was performed with a dilating catheter (10 mm × 4 cm; Boston Scientific, Hurricane, USA), and ballooning time was 3 minutes. In the presence of continuous oozing after initial balloon inflation, balloon tamponade was repeated and the duration was increased further to 5 minutes. The sphincterotomy site was observed for 1–3 minutes to ensure no further bleeding.

#### Definition

Severity of bleeding was classified as mild (with endoscopic evidence of bleeding, with a hemoglobin decrease <3 g/dL, without any need for blood transfusion), moderate (requires blood transfusion of 4 units or less, without any need for angiographic or surgical intervention), or severe (requires >4 units of blood transfusion or intervention) [11].

Onset of bleeding was classified as immediate (hemorrhage at the time of sphincterotomy) or delayed (hemorrhage after sphincterotomy and manifested by melena, hematemesis, or hematochezia associated with a decrease in hemoglobin level).

Procedure-induced pancreatitis was defined as new or worsened abdominal pain and a serum concentration of pancreatic enzymes (amylase or lipase) that was three or more times the upper limit of normal. Procedure-induced cholangitis was defined as fever, abdominal pain, and further elevation of the bilirubin level from baseline. Continuation of preexisting acute pancreatitis and cholangitis were not included as complications.

#### Statistical analysis

Descriptive statistics for continuous variables were calculated and reported as mean±standard deviation (SD). The categorical variables were described using frequency distributions and were reported as n (%). P values were based on analysis of variance (ANOVA) for continuous variables and on Chi-squared test for categorical variables. Logistic regression analysis was performed to identify the risk factors of post-ES bleeding. Statistical analyses were performed using Statistical Package for the Social Science (SPSS) for Windows, version 12.0 (SPSS, Chicago, IL, USA). Tests were two-tailed, with a significance level of 0.05.

## Results

Five hundred thirteen patients underwent ES, and bleeding episodes occurred in 65 (12.6%) patients. The clinical characteristics and risk factors of post-ES bleeding are outlined in Table 1. There were no significant differences in age, sex distribution, and indications for ERCP between the two groups. The risk factors of univariate analysis associated with post-ES bleeding were liver cirrhosis, end-stage renal disease, previous anti-platelet agent use, and duodenal ulcer. In terms of laboratory parameters, higher serum bilirubin and creatinine levels were associated with post ES-bleeding. The complications of pancreatitis and cholangitis were higher in the bleeding group.

**Table 1:** Clinical characteristics of patients in the post-endoscopic sphincterotomy bleeding and non-bleeding groups.

Characteristics	Bleeding n = 65 (%)	Non-bleeding n = 448 (%)	P-value
**Gender (male)**	34 (52.3)	217(48.4)	0.571^†^
**Age (yr) (range)**	62±18.4(8–94)	63±16.1(21–98)	0.250^§^
**Reason of ES**			
CBD stone	40(61.5)	264(58.9)	0.664^†^
Jaundice	12(18.4)	87 (19.4)	0.848^†^
Pancreatitis	1(1.5)	17 (3.8)	0.354^†^
Sphincter of Oddi dysfunction	6(9.2)	63 (14.1)	0.286^†^
Malignancy	4(6.1)	13 (1.2)	0.172^†^
Pre-cut	2(3.1)	4 (0.9)	0.127^†^
**Comorbid conditions**			
Liver cirrhosis	8(7.7)	15(3.3)	0.001^†^
ESRD	6(9.2)	10(2.2)	0.003^†^
Anticoagulant agent use	1(1.5)	5(1.1)	0.767^†^
Antiplatelet agent use	14(21.5)	25(5.6)	<0.001^†^
CBD dilation	46(70.8)	294(65.6)	0.333^†^
CBD stone size(cm)	0.56±0.54	0.46±0.47	0.166^§^
Duodenal ulcer	20(30.8)	83(18.5)	0.022^†^
JPD	24(36.9)	151(33.7)	0.626^†^
**Laboratory parameters**			
Total bilirubin (mg/dL)	5.86±7.28	3.77±4.23	0.037^§^
PT INR	1.10±0.15	1.08±0.10	0.149^§^
Platelet(10^3/uL)	208.6±79.9	228.8±91.0	0.252^§^
Creatinine (mg/dL)	1.40±1.72	1.02±0.76	0.021^§^
**Complication**			
Cholangitis	13 (28.8)	11 (2.5)	<0.001^†^
Pancreatitis	15 (23)	30 (6.7)	<0.001^†^

Abbreviations: ES, endoscopic sphincterotomy; ESRD, end-stage renal disease; CBD, common bile duct; JPD, juxtapapillary diverticulum,; PT INR, prothrombin ratio and international normalized ratio

P value was determined using ANOVA^§^ or Chi-squared test^†^.

Variables associated with risk factors of post-ES bleeding in the multivariable analysis are presented in Table 2. Previous anti-platelet agent use was an important risk factor of relapse (OR: 4.95, 95% CI: 2.25–10.90, P<0.001). The other three parameters, liver cirrhosis, end-stage renal disease, and duodenal ulcer, remained statistically significant.

**Table 2:** Multivariate analysis of potential risk factors for post-endoscopic sphincterotomy bleeding.

Variable	Odds ratio	95% Conf. Interval	P-value
**Reason of ES**			
CBD stone	0.55	0.13–2.28	0.411
Jaundice	0.62	0.14–2.80	0.541
Pancreatitis	0.27	0.02–3.33	0.314
Sphincter of Oddi dysfunction	0.27	0.05–1.69	0.164
Malignancy	1.43	0.24–8.78	0.694
Pre-cut	2.86	0.31–26.50	0.354
**Comorbid conditions**			
Liver cirrhosis	3.10	1.11–8.60	0.029
ESRD	3.55	1.07–11.76	0.038
Anticoagulant agent use	0.94	0.09–9.83	0.962
Antiplatelet agent use	4.95	2.25–10.90	<0.001
CBD dilation	1.24	0.67–2.32	0.486
Duodenal ulcer	2.06	1.11–3.87	0.023
JPD	0.82	0.51–1.71	0.821

Abbreviations: ES, endoscopic sphincterotomy; ESRD, end-stage renal disease; CBD, common bile duct; JPD, juxtapapillary diverticulum

P value was determined using logistic regression.

Among 65 patients with post ES-bleeding, immediate and delayed bleeding were noted in 45 (69.2%) and 20 (30.8%) patients, respectively. S1 Table lists their demographic, laboratory, and clinical data. All immediate bleeding patients received proton pump inhibitor (esomeprazole sodium, 40 mg, twice a day for 3 days) to prevent delayed bleeding. Five (11.1%) of 45 patients had re-bleeding after initial endoscopic hemostasis with epinephrine spray for immediate post-ES bleeding.

Delayed bleeding occurred 1–7 days (mean, 2.5 days) following the procedures. Among the 20 patients who had delayed bleeding, 4 (20%) had severe bleeding, which was more severe than those with immediate bleeding. There were no significant differences in age, sex distribution, comorbidity of end-stage renal disease, previous anti-platelet/anti-coagulant agent use, duodenal ulcer, and laboratory values between the two groups. The presence of liver cirrhosis was associated with higher risk of delayed post-ES bleeding. However, when stratified in accordance with the Child-Pugh classification of cirrhosis, no statistically significant difference was found between the two groups. Epinephrine spray was a common procedure used in patients with immediate bleeding, whereas epinephrine injection was commonly applied in those with delayed bleeding.

Among the 20 patients with delayed bleeding (S2 Table), 12 (60%) patients received endoscopic intervention. Seven patients received a side-viewing and five patients received end-viewing endoscope. During endoscopy, four patients had active oozing, four patients had adherent clot, two patients had non-bleeding visible vessel, and two patients had ulcer. Hemostatic therapy was done in seven patients, but two patients failed to achieve initial hemostasis. These two patients recovered after supportive treatment. One patient with liver cirrhosis and uremia died of post-ES bleeding–related multiple organ failure. No patient required angiography or surgery for delayed bleeding.

Outcomes of different endoscopic hemostatic therapies are listed in Table 3. The use of epinephrine spray was associated with higher complication of cholangitis, whereas epinephrine injection and thermocoagulation were associated with higher complication of pancreatitis. The outcome of the endoclip and balloon dilation group was not statistically different.

**Table 3:** Comparison of complication between the different hemostatic treatment.

Characteristics	Pancreatitis N = 15 (%)	Non-pancreatitis N = 50(%)	P-value	Conlangitis N = 52(%)	Non- conlangitis n = 13(%)	P-value
Epinephrine spray	13(86.6)	29(58)	0.364	35(67.3)	7(53.8)	0.042
Epinephrine injection	3(20)	2(4)	0.041	3(5.8)	2(15.4)	0.245
Thermocoagulation	7(46.7)	10(20)	0.039	15(28.8)	2(15.4)	0.323
Endoclip	1(6.7)	0(0)	0.066	1(1.9)	0 (0)	0.614
Ballon dilation	0(0)	5(10)	0.202	5(9.6)	0 (0)	0.245

Compared with patients receiving endoscopic hemostatic therapy versus no endoscopic therapy (Table 4), the severity of bleeding was not statistically different. However, endoscopic hemostatic therapy was performed more frequently in the immediate bleeding group than in the delayed bleeding group.

**Table 4:** Comparison of post-endoscopic sphincterotomy bleeding between endoscopic interventional and non-interventional therapy groups.

Characteristics	Interventional	Non-interventional	P-value
Timing of bleeding			
Immediate	44	1	<0.001
Delayed	12	8	
Severity of bleeding			
Mild	43	11	0.977
Moderate	4	1	
Severe	5	1	

## Discussion

The endoscopic approach for post-ES bleeding is similar to that for peptic ulcer bleeding; however, there is no optimal endoscopic hemostasis because it is challenging to localize the bleeder or to administer interventions through a side-viewing endoscope. This study revealed that post-ES bleeding was associated with higher complications of cholangitis and pancreatitis and that each therapeutic modality had different complications. The post-ES bleeding rate was 12.6% in this study, which was higher than those seen in previous studies [1, 2]. The difference may result from the different definitions of bleeding, and we included all severities of bleeding seen at the time of endoscopy.

The risk factors of post-ES bleeding were well studied [1, 7, 12, 13], including any degree of bleeding during the procedure, presence of coagulopathy or thrombocytopenia, initiation of anticoagulant therapy within 3 days after ES, liver cirrhosis, dilated common bile duct, periampullary diverticulum, precut sphincterotomy, and relatively low case volume on the part of the endoscopist (one sphincterotomy per week or fewer). Our study showed that the predicting factors associated with post-ES bleeding were liver cirrhosis, end-stage renal disease, previous anti-platelet drug use, and duodenal ulcer. The higher-risk procedure of precut sphincterotomy for patients with difficult cannulation did not yield statistical significance as in previous publications, which might be related to the technique being performed less in our hospital.

Recently, the European consensus recommended the discontinuation of clopidogrel 5 days before the procedure and continuation of aspirin use for high-risk endoscopic procedures such as ES [14]. However, an Asian study revealed an increased risk of post-ES bleeding even when aspirin was withheld for 1 week (10% vs. 4%, P = 0.03) [15]. There was an approximately twofold increased risk of gastrointestinal bleeding with aspirin usage in a systemic study [16]. Withholding antiplatelet agent 3 days before the procedure also posed a risk of bleeding in this study. Therefore, controversy remains regarding the appropriate use of antiplatelet agent, particularly in Asian patients with other risk factors for post-ES bleeding.

Several new endoscopic hemostasis technologies have been developed recently. Cap-assisted end-viewing endoscopy using endoclips successfully controlled the hemorrhage in 90% cases, resolving the problem of kink when passing through the elevator of the instrument channel through a side-viewing endoscopy [17, 18]. Fully covered metallic biliary stents have also been applied in post-ES bleeding and have afforded good outcomes [19]. However, higher cost and the need for repeat ERCP for the removal of the stent limit the widespread application of this method. Fibrin glue injection was another effective endoscopic hemostatic therapy for refractory post-ES bleeding, but nasobiliary and nasopancreatic drainage for the diagnosis of intraductal fibrin clots and prevention of complication of outflow obstruction was needed [20].

Complications associated with endoscopic hemostasis may occur, but it may be difficult to distinguish which of these were related to the ERCP itself and which were related to the treatment of bleeding. A previous study revealed that the complication rate was higher in the group that underwent endoscopic hemostasis (7.3% vs. 5.1%) [10]. In this study, the risk of cholangitis was higher in the post-ES bleeding group (28.8% vs. 2.8% P<0.001) and with the use of epinephrine spray (67.3% vs. 53.8% P = 0.042), which may be related to biliary reflux of duodenal chyme after ES and epinephrine spray, causing ascending bile duct infection. Meanwhile, when the hemorrhage occurs more slowly, blood and bile do not mix owing to their different specific gravity and surface tension. If the rate of blood clot formation and the temporary effect of blood vessel constriction by epinephrine spray exceed the fibrinolytic capacity of the bile, the resultant clots obstruct the bile duct and lead to cholangitis [21]. Furthermore, recurrent post-ES bleeding and repeated hemostasis procedure wound also increase the rate of cholangitis [12].

ES does not appear to add significant independent risk of pancreatitis to ERCP [22]. However, post-ES bleeding was associated with higher rate of pancreatitis (23% vs. 6.7%, P<0.001) in this study. The risk of pancreatitis was higher in the therapeutic group who underwent epinephrine injection and electrocoagulation, which may due to the procedure not being an accurate target and thus papillary trauma occurred later. Furthermore, blood clots refluxed into and obstructed the pancreatic duct wound, which also increased the risk of acute pancreatitis [23].

Recently, a study showed that mild post-ES bleeding could be managed with proton pump inhibitors because this drug could promote thrombus formation and stop bleeding from small vessels [13]. As for larger blood vessel damage or arterial hemorrhage, pharmacological treatment combined with endoscopic treatment is necessary [13]. In this study, the higher rate of endoscopic interventional therapy for immediate post-ES bleeding might relate to better localization of bleeding point and prevention of delayed bleeding. Only 60% of the patients with delayed bleeding received endoscopic evaluation, and the successful hemostasis rate was 71.4%. Nearly 65% of delayed bleeding patients had ceased bleeding without endoscopic hemostatic treatment. Therefore, suitable knowledge of management of different severities of bleeding and complication of therapy are important.

### Limitations

First, this small sample size of this study and its retrospective design might have led to selection bias in choosing the management of bleeding. Second, 69.2% (45/65) patients had immediate post-ES bleeding. Most endoscopists believed immediate bleeding may be self-limiting and those who responded to epinephrine spray may stop spontaneously with conservative treatment [1]. These observations deserve further evaluation in a randomized study. Third, endoscopic hemostasis therapy depended on the endoscopist’s experience. In practice, emergency endoscopy is not always performed by an experienced ERCP endoscopist. The prolonged endoscopic hemostatic time, overtreatment of immediate bleeding, and non-routine usage of prophylactic antibiotic or rectal indomethacin might have increased the complication rate in this study.

## Conclusions

Post-ES bleeding and endoscopic hemostasis wound increase the complication of pancreatitis and cholangitis. Realizing the effectiveness of each therapeutic modality and appropriate management of various degrees of bleeding are important.

## Supporting information

S1 Table Clinical characteristics of patients in the immediate and delayed post-endoscopic sphincterotomy bleeding groups. (DOCX)

S2 Table Endoscopic features and therapy in the delayed post-endoscopic sphincterotomy bleeding. (DOCX)
