# Supplementary material for: Clinical endoscopic management and outcome of post-endoscopic sphincterotomy bleeding
Source: PLoS One. 2017 May 17;12(5):e0177449. doi: 10.1371/journal.pone.0177449 (PMC5435171; doi:10.1371/journal.pone.0177449)
Supplement: S1 Table — (DOCX) [file pone.0177449.s001.docx]

**S1 Table.** Clinical characteristics of patients in the immediate and delayed post-endoscopic sphincterotomy bleeding groups

| **Characteristics** | **Immediate bleeding n=45(%)** | **Delayed bleeding**  **N=20(%)** | **P-value** |
| --- | --- | --- | --- |
| **Gender (male)** | 26(57.8) | 8(40.0) | 0.185^†^ |
| **Age(yr) (range)** | 59±18.9(8-89) | 67±16.6(33-94) | 0.542^§^ |
| **Severity of bleeding** | | | |
| Mild | 41(91.1) | 13(65.0) | 0.034^†^ |
| Moderate | 2(4.4) | 3(15.0) |  |
| Severe | 2(4.4) | 4(20.0) |  |
| **Comorbid conditions** | | | |
| Liver cirrhosis | 3(6.7) | 5(25) | 0.038^†^ |
| Child-Pugh A | 1(2.2) | 2(10) | 0.168^†^ |
| Child-Pugh B | 2(4.4) | 2(10) | 0.390^†^ |
| Child-Pugh C | 0(0) | 1(5) | 0.131^†^ |
| ESRD | 3(6.7) | 3(15) | 0.248^†^ |
| Anticoagulant agent | 1(2.2) | 0(0) | 0.502^†^ |
| Antiplatelet agent | 9(20) | 5(25) | 0.651^†^ |
| CBD dilation | 32(71.1) | 15(75) | 0.746^†^ |
| CBD stone size(cm) | 0.53±0.45 | 0.65±0.70 | 0.479^§^ |
| Duodenal ulcer | 16(35.6) | 4(20) | 0.210^†^ |
| JPD | 16(35.6) | 8(40) | 0.732^†^ |
| **Laboratory parameters** | | | |
| Total bilirubin (mg/dL) | 6.16±8.24 | 5.25±4.60 | 0.496^§^ |
| PT INR | 1.11±0.17 | 1.07±0.09 | 0.455^§^ |
| Platelet(10^3/uL) | 214.9±77.7 | 194.5±82.7 | 0.477^§^ |
| Creatinine (mg/dL) | 1.23±1.44 | 1.78±2.16 | 0.579^§^ |
| **Treatment method** | | | |
| Epinephrine spray | 38(84.4) | 4(20) | <0.001^†^ |
| Epinephrine injection | 1(2.2) | 4(20) | 0.013^†^ |
| Thermocoagulation | 12(26.7) | 5(25) | 0.888^†^ |
| Endoclip | 0(0) | 1(5) | 0.131^†^ |
| Ballon dilation | 5(11.1) | 0(0) | 0.121^†^ |

Abbreviations: ES, endoscopic sphincterotomy ;ESRD, end-stage renal disease; CBD, common bile duct; JPD, juxtapapillary diverticulum,; PT INR, prothrombin ratio and international normalized ratio

P value was determined using ANOVA^§^ or Chi-squared test^†^.
