# Supplementary material for: Clinical endoscopic management and outcome of post-endoscopic sphincterotomy bleeding
Source: PLoS One. 2017 May 17;12(5):e0177449. doi: 10.1371/journal.pone.0177449 (PMC5435171; doi:10.1371/journal.pone.0177449)
Supplement: S2 Table — (DOCX) [file pone.0177449.s002.docx]

**S2 Table.** Endoscopic features and therapy in the delayed post-endoscopic sphincterotomy bleeding

| **Characteristics** | **Delayed bleeding n=20 (%)** |
| --- | --- |
| **Endoscopic intervention** | |
| End-viewing endoscopy | 7(35) |
| Side-viewing endoscopy | 5(25) |
| **Bleeding stigmata** | |
| Oozing | 4(20) |
| Adherent clot | 4(20) |
| Non-bleeding visible vessel | 2(10) |
| Ulcer | 2(10) |
| **Cease bleeding** | |
| Yes | 5(25) |
| No | 2(10) |
| Spontaneous | 13(65) |
